# Prediction of the Potential Distribution of *Cirsium vulgare* in China Under Climate Change Scenarios Based on Its First Recorded Occurrences in Southern China

**DOI:** 10.1002/ece3.74028

**Published:** 2026-07-19

**Authors:** Rongtao Hu, Wei Zhang, Zhijie Wang, Qiurui Li, David Roy Clements, Michael Denny Day, Shicai Shen

**Affiliations:** ^1^ School of Agriculture Yunnan University Kunming China; ^2^ Key Laboratory of Prevention and Control of Biological Invasions, Ministry of Agriculture and Rural Affairs of China, Agricultural Environment and Resource Research Institute Yunnan Academy of Agricultural Sciences Kunming China; ^3^ Key Laboratory of Green Prevention and Control of Agricultural Transboundary Pests of Yunnan Province, Agricultural Environment and Resource Research Institute Yunnan Academy of Agricultural Sciences Kunming China; ^4^ School of Ethnology and Sociology Minzu University of China Beijing China; ^5^ Department of Biology Trinity Western University Langley British Columbia Canada; ^6^ Department of Primary Industries Brisbane Queensland Australia

**Keywords:** biological invasion, dual environmental response pattern, inverse elevational shift, MaxEnt model

## Abstract

*Cirsium vulgare* (Asteraceae) is an invasive plant species whose distribution is expanding globally. It was recently recorded in Yunnan Province, China, far south of its previous known range in China, necessitating a timely invasion risk assessment. This study aimed to provide a more comprehensive assessment of its potential distribution in China under climate change scenarios. We used an optimized MaxEnt model combined with a Multivariate Environmental Similarity Surface (MESS) analysis, based on current global occurrences and 52 newly collected field occurrence records from Yunnan Province. The model demonstrated robust predictive performance (AUC = 0.971) and identified temperature seasonality (Bio4, contribution rate 42.8%) and precipitation seasonality (Bio15, 20.0%) as the two main influencing factors. The species exhibited a dual environmental response pattern that would allow it to adapt to mild, low‐seasonality settings of southwestern highlands, while also enduring the harsh, strongly continental climate of the northwestern inland. Thus, facilitating a multi‐center distribution pattern including Yunnan, Xinjiang and Taiwan. Under future climate change scenarios, the model showed *C. vulgare* displayed a rare inverse elevational shift. There was a significant centroid shifting northwestward and a sharp decline in elevation from the current high‐altitude mountainous region (4900 m) to the edge of the Qaidam Basin (3100 m). This suggests that *C. vulgare* populations are potentially able to track high‐seasonality climates to offset warming impacts caused by climate change scenarios. Although the total suitable area of *C. vulgare* in China is projected to contract in the future, the Yunnan–Guizhou Plateau and Xinjiang's Ili River Valley remain stable climate refugia. Therefore, this study recommends considering these core distribution areas as primary priorities for prevention and control of *C. vulgare* and highlights the colonization risk to oasis agricultural regions via the northwestern dispersal corridors.

## Graphical abstract

This study aimed to determine its potential distribution under climate change scenarios using a MaxEnt model and Multivariate Environmental Similarity Surface (MESS) analysis based on the new Yunnan records and current global occurrences. This study elucidates the invasion pattern of *C. vulgare* and provides a scientific basis for understanding the mechanisms of niche shifts in high‐altitude invasive plants in response to climate change.

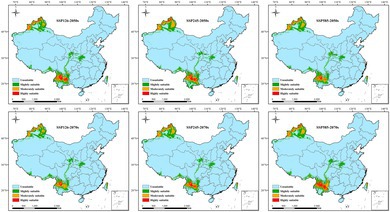

## 1 Introduction

Against the backdrop of intensifying global climate change and human activity, biological invasions have emerged as a critical challenge, threatening global biodiversity and agricultural sustainability (Ojija et al. 2019a, 2019b; Pyšek et al. 2020; Diagne et al. 2021; Peller and Altermatt 2024). Climate change directly facilitates the colonization and geographical expansion of alien species, particularly those with broad ecological tolerances, enabling them to breach historical environmental barriers into high‐altitude or high‐latitude regions (Pauchard et al. 2009; Bellard et al. 2013). Consequently, accurately predicting the potential suitable habitats of invasive plants under future climate scenarios is crucial for developing early warning mechanisms (Guisan et al. 2013).

A prominent example of a highly adaptable invasive species capitalizing on such environmental shifts is *Cirsium vulgare* (Savi) Ten. (Asteraceae). Native to Europe, Western Asia, and North Africa, this biennial or short‐lived perennial herb has spread worldwide due to its prodigious reproductive capacity, effective wind dispersal, and strong adaptability to disturbed habitats (Forcella and Wood 1986; Doucet and Cavers 1997). Where it invades, *C. vulgare* causes severe ecological and economic damage by displacing native flora, reducing pasture carrying capacity, and serving as an intermediate host for crop pests (Cripps et al. 2011; Román et al. 2021; Bureš et al. 2024). Furthermore, its spiny morphology makes manual eradication difficult, while its allelopathic potential actively facilitates its continued expansion (Klinkhamer and De Jong 1993; Zheng, Zhan, et al. 2026).

In China, *C. vulgare* has long been considered to be restricted primarily to the northwestern Xinjiang Uygur Autonomous Region (XUAR), an area dominated by a typical temperate continental climate (Editorial Committee of the Flora of China of Chinese Academy of Sciences 1987). However, extensive field surveys conducted between 2022 and 2024, documented naturalized populations of *C. vulgare* for the first time far to the south, in Yunnan Province (Zheng, Zhan, et al. 2026). The species has widely invaded farmlands, wastelands, and managed landscapes across Kunming, Chuxiong, Yuxi, and Qujing. This unexpected emergence in a subtropical highland region challenges the prevailing view of the climatic limitations of *C. vulgare* , suggesting that *C. vulgare* possesses more complex environmental adaptation mechanisms than previously recognized, and that its national invasion risk has been underestimated.

To address this risk, Species Distribution Models (SDMs) such as the Maximum Entropy (MaxEnt) algorithm, offer a robust framework for predicting invasion dynamics using presence‐only data (Phillips et al. 2006). However, existing distribution studies on *C. vulgare* have largely been limited to local scales or historical records, failing to incorporate the newly discovered Yunnan populations or future climate projections. Furthermore, traditional modeling often relies on default software parameters, which can lead to model overfitting and limit transferability in non‐native ranges (Warren and Seifert 2011; Radosavljevic et al. 2014).

Therefore, this study presents the first national‐scale modeling effort for *C. vulgare* in China. By integrating the newly discovered Yunnan occurrences with global database records, we employed a parameter‐optimized MaxEnt model, coupled with Multivariate Environmental Similarity Surfaces (MESS) analysis (Elith et al. 2010; Cobos et al. 2019). Our specific objectives were to: (1) construct a high‐precision potential distribution model for China; (2) quantify the dominant environmental factors driving its dual adaptation across vast northwestern and southwestern environmental gradients; and (3) simulate the spatiotemporal dynamics and centroid migration trajectories of its suitable areas under future climate change scenarios.

## 2 Materials and Methods

### 2.1 Occurrence Data Collection and Spatial Filtering

**FIGURE 1:**
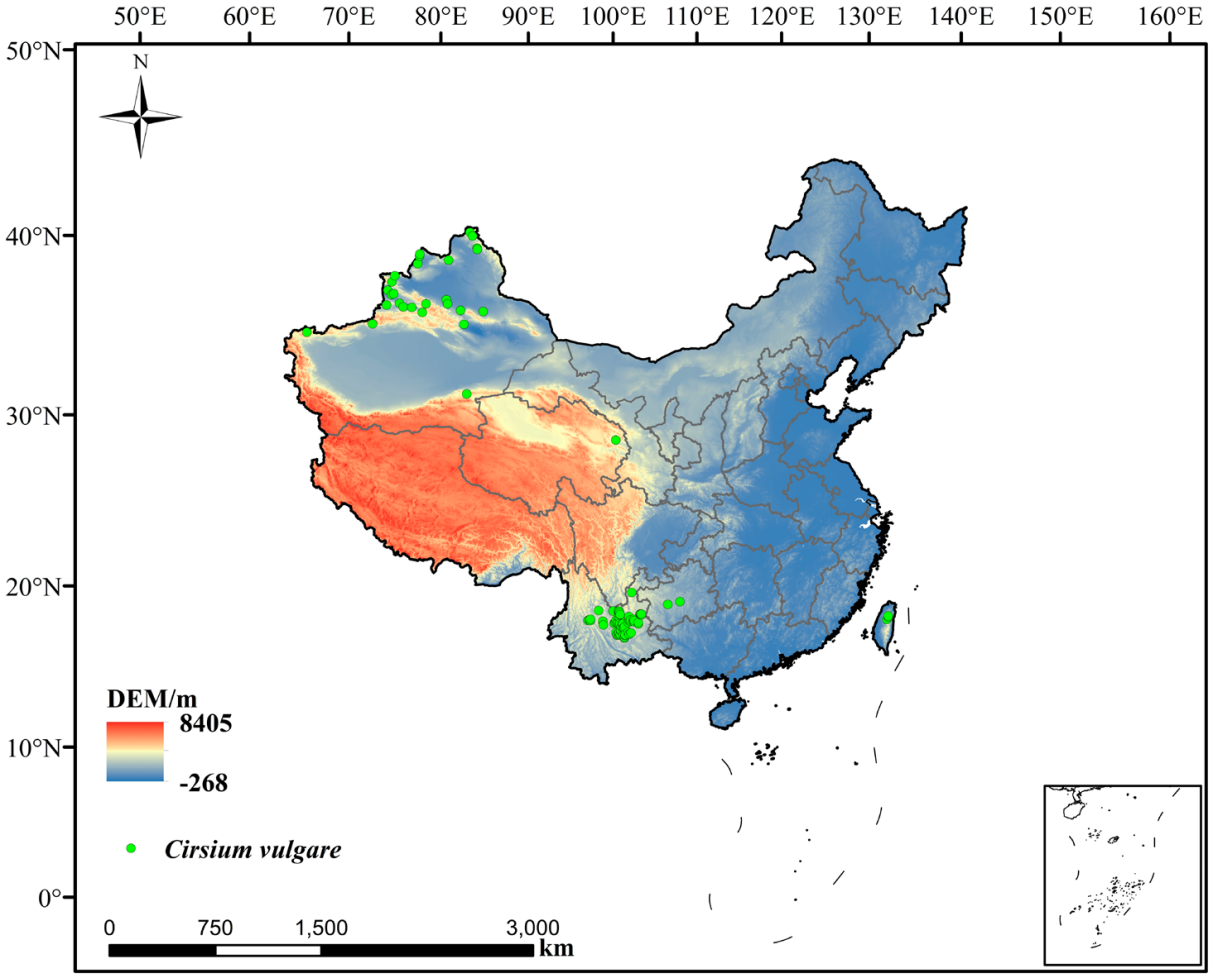
Distribution of sampling sites for *C. vulgare* in China.

In this study, 52 occurrence records of *C. vulgare* were obtained through extensive field surveys conducted in 2022 across multiple regions in Yunnan Province, China, specifically including Kunming City, Chuxiong Yi Autonomous Prefecture, Yuxi City, and Qujing City. Additionally, 53 occurrence records (mainly located in XUAR) were collected from the Global Biodiversity Information Facility (GBIF, https://doi.org/10.15468/dl.qqx4cy, accessed on 06 August 2024) and the Chinese Virtual Herbarium (CVH, https://www.cvh.ac.cn/spms/list.php?taxonName=Cirsium+vulgare&offset=0, accessed on 06 August 2024), giving a total of 105 occurrence records of *C. vulgare* in China (Figure 1). To reduce spatial sampling bias and mitigate spatial autocorrelation caused by clustered occurrences (Boria et al. 2014), we spatially filtered the dataset using the trim duplicate occurrences function in ENMTools (Warren et al. 2010). Specifically, we retained only one occurrence record within each 2.5′ × 2.5′ grid cell. Ultimately, 97 *C. vulgare* occurrence points were retained for MaxEnt modeling.

### 2.2 Environmental Predictors: Acquisition and Screening

**FIGURE 2:**
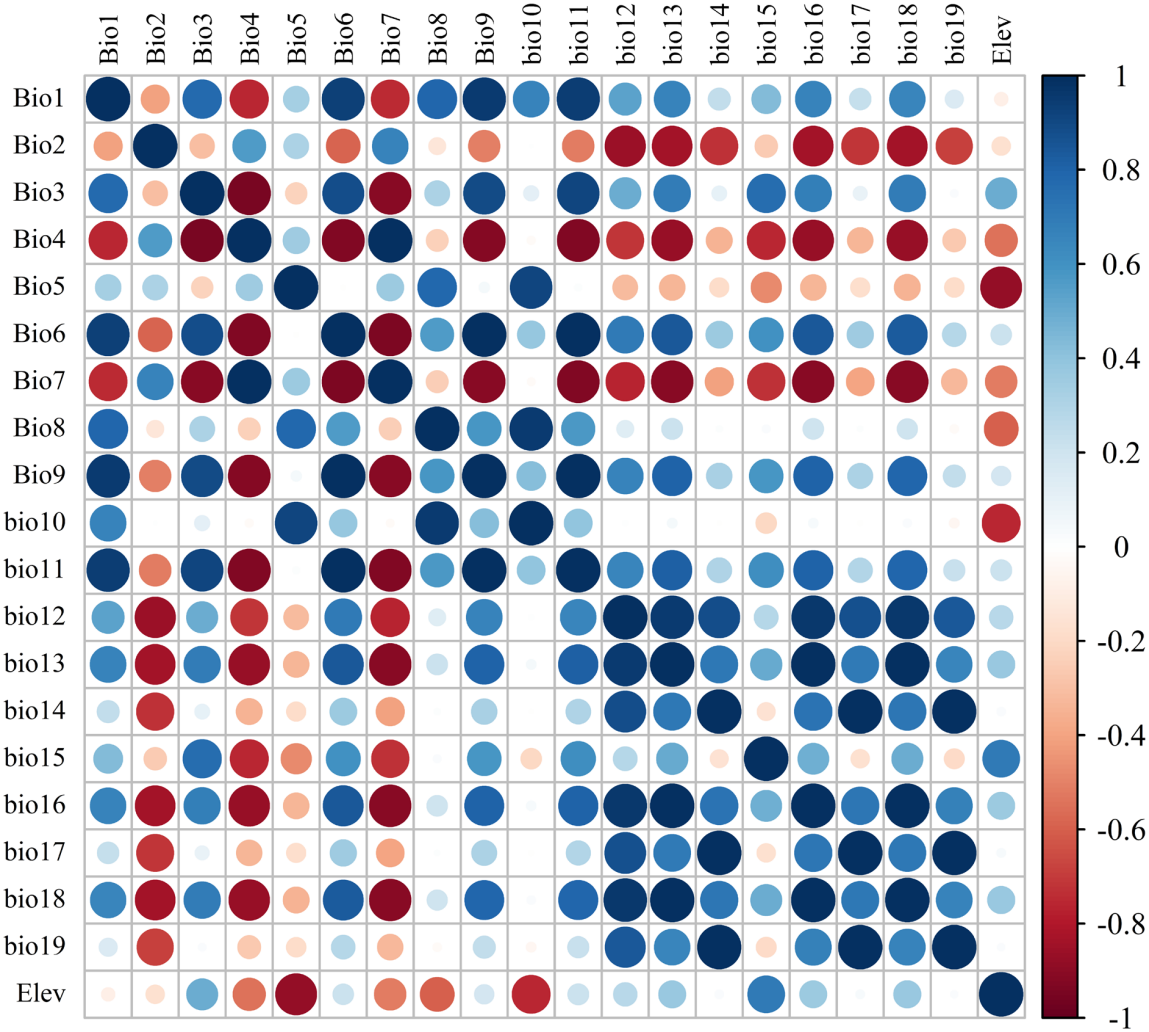
Correlation test of environmental variables for *C. vulgare* .

Environmental predictors, comprising 19 bioclimatic variables and one topographical factor (elevation), were acquired from the WorldClim v2.1 database (Fick and Hijmans 2017; https://worldclim.org/). These covered three time periods: the current period (1970–2000) and future periods including the 2050s (2041–2060) and 2070s (2061–2080). Future climate projections were derived from the Beijing Climate Center Climate System Model (BCC‐CSM2‐MR) under CMIP6, which has demonstrated strong performance in capturing regional climate variability and climate extremes and has been widely applied in species distribution studies in China (Wu et al. 2019). We selected three Shared Socioeconomic Pathways (SSPs) to represent different greenhouse gas emission scenarios: SSP126 (low emissions), SSP245 (intermediate emissions), and SSP585 (high emissions). To meet accuracy and computational requirements, the spatial resolution for all environmental variables was set to 2.5 arc‐minutes. To minimize overfitting caused by multicollinearity and to improve prediction accuracy (Dormann et al. 2013), a Pearson correlation analysis was performed on the 20 environmental variables using SPSS 20 software (Figure 2). Variable selection was based on considering both pairwise correlations (|*r*| > 0.8) and preliminary variable importance (percent contribution) from an initial MaxEnt run. Variables that were strongly correlated and showed lower contributions were progressively eliminated (Yang et al. 2013). Ultimately, five dominant environmental variables were selected for the final prediction model of *C. vulgare* (Table 1).

**TABLE 1:** Preselected environmental variables and final variables used for model prediction of *C. vulgare* .

Field	Description	Unit
Bio1	Annual mean temperature	°C
**Bio2**	**Mean diurnal temperature range**	**°C**
Bio3	Isothermality	—
**Bio4**	**Temperature seasonality**	**—**
Bio5	Maximum temperature of warmest month	°C
Bio6	Minimum temperature of coldest month	°C
Bio7	Temperature annual range	°C
Bio8	Mean temperature of wettest quarter	°C
Bio9	Mean temperature of driest quarter	°C
Bio10	Mean temperature of warmest quarter	°C
Bi11	Mean temperature of coldest quarter	°C
Bio12	Annual precipitation	mm
Bio13	Precipitation of wettest month	mm
**Bio14**	**Precipitation of driest month**	**mm**
**Bio15**	**Precipitation seasonality**	**mm**
Bio16	Precipitation of wettest quarter	mm
Bio17	Precipitation of driest quarter	mm
Bio18	Precipitation of warmest quarter	mm
Bio19	Precipitation of coldest quarter	mm
**Elev**.	**Elevation**	**m**

*Note:* Bold environmental variables indicate the final variables used for model construction.

### 2.3 Model Parameter Optimization, Construction and Evaluation

The predictive performance of the MaxEnt model is primarily influenced by two key parameters: the Regularization Multiplier (RM) and Feature Classes (FC) (Radosavljevic et al. 2014). To determine the optimal parameters, the kuenm package in R (version 3.6.3) was used to generate candidate models based on a full factorial combination of RM and FC (Zhang et al. 2025). The RM range was set from 0.1 to 4.0, with a step size of 0.1, totaling 40 options. Additionally, 29 FC combinations involving linear (L), quadratic (Q), product (P), threshold (T), and hinge (H) features were selected for testing: L, Q, P, T, H, LQ, LP, LT, LH, QP, QT, QH, PT, PH, TH, LQP, LQT, LQH, LPT, LPH, QPT, QPH, QTH, PTH, LQPT, LQPH, LQTH, LPTH, and LQPTH. The kuenm package screened significant models with an omission rate ≤ 5% from 1160 candidate models. Subsequently, the configuration with a Delta AICc value of 0 was selected as the optimal parameter set for MaxEnt modeling (Cobos et al. 2019).

The filtered occurrence data (97 records) and the five selected environmental variables were imported into MaxEnt v3.4.4, and the model was configured using the optimized parameters described above. The dataset was randomly divided into two parts: 75% for model training and the remaining 25% for testing and validation (Phillips and Dudík 2008). The maximum number of background points was set to 10,000, and these points were randomly drawn from the entire terrestrial area of China, which was defined as the background extent for model calibration. The maximum number of iterations was set to 500 to ensure convergence. The calculation results were obtained by averaging the logistic output values from 10 runs, with the repetition category set to subsample (Phillips and Dudík 2008). In the environmental parameter settings, the jackknife test was used to evaluate the contribution of environmental variables and identify the dominant factors affecting the distribution of *C. vulgare* . To measure the performance of the MaxEnt model, the area under the curve (AUC) of the receiver operating characteristic (ROC) curve was used to evaluate the discriminatory ability of the model (Escobar 2020; Gebrewahid et al. 2020). An average AUC value exceeding 0.8 indicates good model performance, while an AUC value exceeding 0.9 indicates excellent performance (Luu et al. 2021; Sun et al. 2020).

### 2.4 Classification of Suitable Areas

To visually display the potential spatial distribution pattern of *C. vulgare* , the average logistic values (logistic prediction probabilities) generated by the MaxEnt model were reclassified using ArcGIS v10.4. Logistic values range from 0 to 1, with higher values indicating higher area suitability (Phillips and Dudík 2008). The Jenks natural breaks method (Jenks 1967) was applied to the logistic output under the current climate scenario to determine classification thresholds. To ensure comparability among current and future projections, the same thresholds derived from the current scenario were applied to all future climate scenarios. Accordingly, the potential distribution areas of *C. vulgare* in China were classified into four grades: unsuitable (0 ≤ *p* ≤ 0.09), slightly suitable (0.09 < *p* ≤ 0.28), moderately suitable (0.28 < *p* ≤ 0.53), and highly suitable (0.53 < *p* ≤ 0.86) (Table 2). This method was selected due to its mature application in classifying spatial data into homogeneous groups, effectively identifying inherent clustering to aid in interpreting species distribution patterns (Shen et al. 2024; Wang et al. 2024).

**TABLE 2:** Thresholds for habitat suitability classification based on Jenks' natural breaks.

Species	Unsuitable	Low suitability	Moderate suitability	High suitability
*C. vulgare*	0–0.0944	0.0944–0.2832	0.2832–0.5327	0.5327–0.8564

### 2.5 Potential Suitable Area Distribution and Centroid Shifts

Based on the Maximum Training Sensitivity plus Specificity (MTSS) criterion (Liu et al. 2005), a threshold of 0.1785 was selected to reclassify the continuous area suitability map of *C. vulgare* generated by the MaxEnt model into a binary map. Using SDMtoolbox (an ArcGIS toolkit implemented in Python) (Brown et al. 2017), the geographic distribution changes of *C. vulgare* under current and future climate scenarios (SSP126, SSP245, SSP585) were calculated and analyzed. Furthermore, the centroid coordinates of suitable areas (representing the core distribution center) were calculated using SDMtoolbox to quantify directional migration (Zhuo et al. 2020). The migration distance was calculated by measuring the displacement of the centroid from the current period to future periods, thereby demonstrating the spatial trajectory of distribution range shifts.

### 2.6 Multivariate Environmental Similarity Surface (MESS) and Most Dissimilar (MoD) Variable Analysis

A Multivariate Environmental Similarity Surface (MESS) analysis was used to assess the similarity between climate conditions at a specific location during a specific time period and the calibration (training) environmental space. It quantifies climate similarity by calculating the similarity score for each environmental variable, with the final MESS value corresponding to the minimum similarity value across all variables. Negative values (*S* < 0) indicate that at least one environmental variable falls outside the range observed in the calibration data, reflecting environmental novelty and potential extrapolation risk, whereas higher positive values (*S* > 0) indicate greater environmental similarity to the calibration space and higher reliability of model predictions (Elith et al. 2010).

The Most Dissimilar (MoD) variable analysis was used to identify key climate factors driving potential changes in suitable areas under different climate scenarios (Elith et al. 2010). In this study, this analysis was integrated into the final model construction and projection phase, implemented automatically via the kuenm R package (Cobos et al. 2019). We enabled the parameter ext_type = “E”, thereby automatically generating MESS layers and associated extrapolation risk analysis results when projecting the model to different climate scenarios.

## 3 Results

### 3.1 Model Optimization and Accuracy Evaluation

**FIGURE 3:**
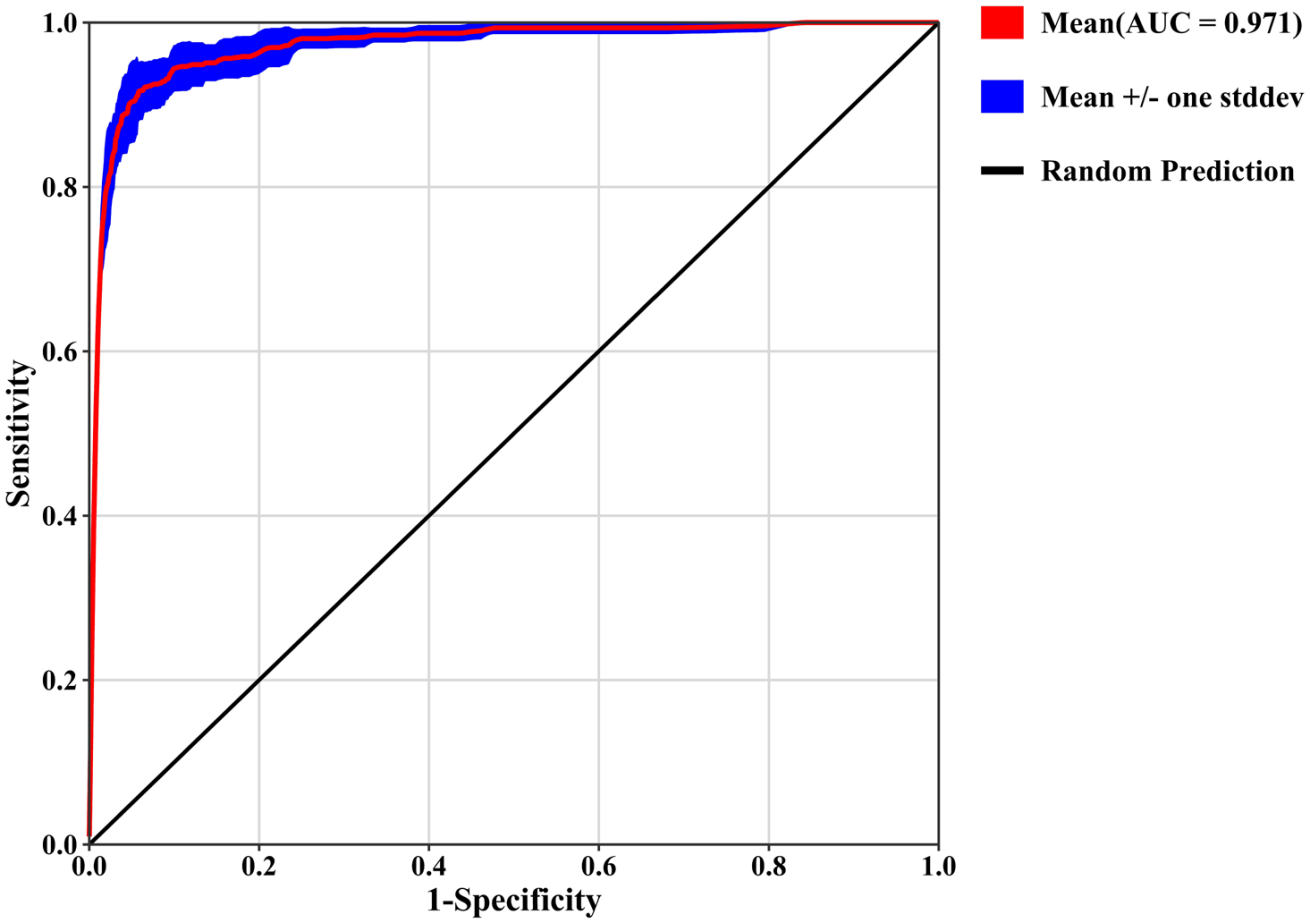
ROC test of the MaxEnt model with optimized parameters.

Under default settings (FC = LQPTH, RM = 1), the Delta AICc value for *C. vulgare* was 92.72. When optimized to FC = QTH and RM = 1.5, the Delta AICc was 0. Parameter optimization resulted in a 31% reduction in omission rates, indicating that the model fit to the known occurrence records was significantly improved (Table 3). Using these optimal settings, the mean AUC value of the MaxEnt model for *C. vulgare* was 0.971 ± 0.009 (Figure 3), indicating that the prediction results are accurate and reliable.

**TABLE 3:** Evaluation results of the MaxEnt model under different parameter settings.

Setting	FC	RM	Delta AICc	Omission rate at 5%
Default	LQPTH	1	92.72	0.13
Optimized	QTH	1.5	0	0.09

### 3.2 Key Environmental Variables Influencing Distribution

Based on a jackknife test, contribution rate and permutation importance from the MaxEnt model, temperature seasonality (Bio4) was identified as the dominant predictor (percent contribution = 42.8%), followed by precipitation seasonality (Bio15, 20.0%) and Elevation (17.9%) (Table 4). These results suggest that the distribution of *C. vulgare* is jointly shaped by seasonal variability in temperature and precipitation, together with topographic effects.

**TABLE 4:** Percent contribution and permutation importance of key environmental variable for *C. vulgare* .

Environmental variable	Contribution rate (%)	Permutation importance (%)
Temperature seasonality (Bio04)	42.8	4.2
Precipitation seasonality (Bio15)	20	45.4
Elevation	17.9	31.7
Precipitation of the driest month (Bio14)	13.7	11.4
Mean diurnal range (Bio02)	5.6	7.4

**FIGURE 4:**
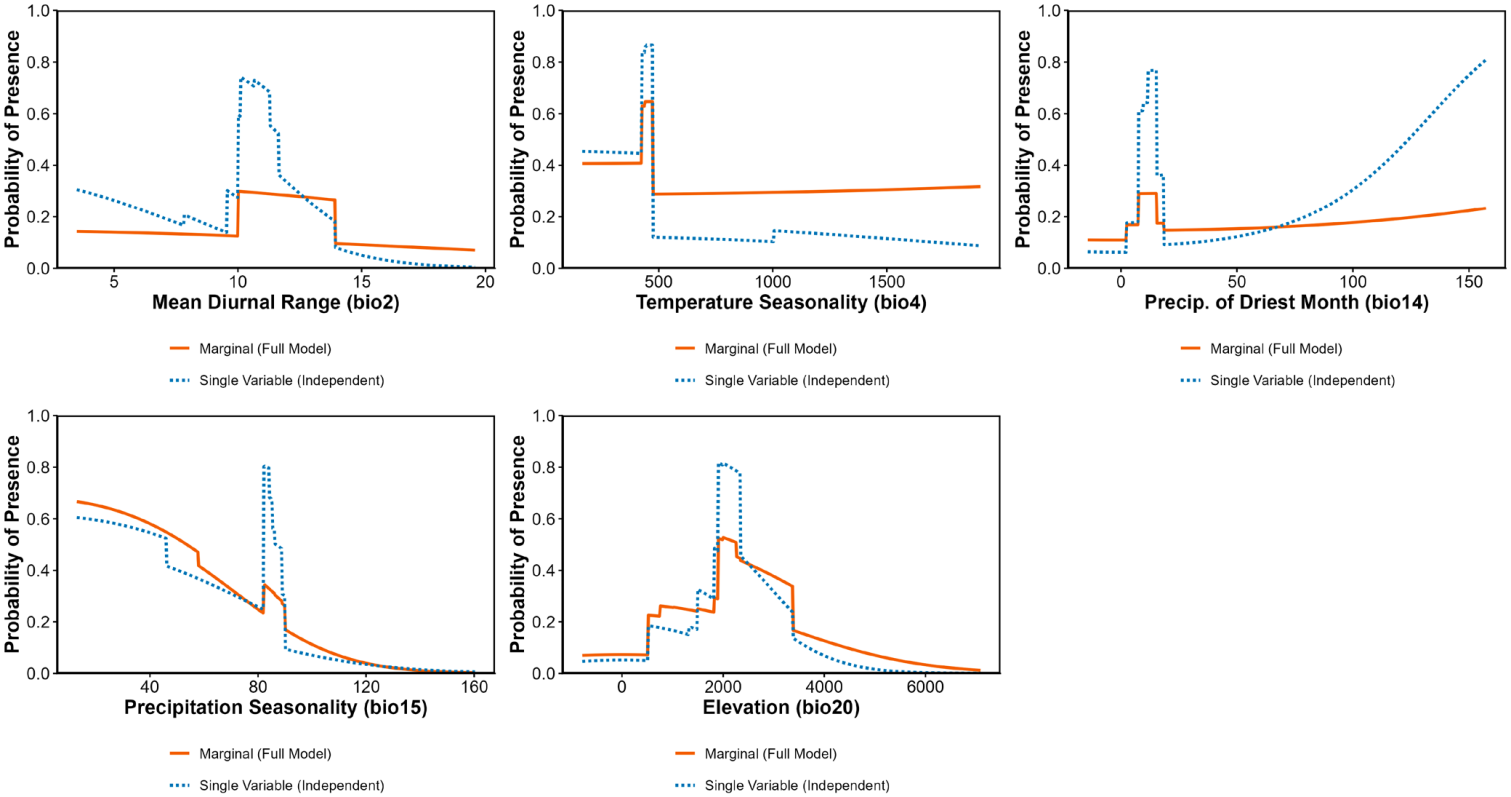
Response curves for environmental predictors of *C. vulgare* . Orange solid lines show marginal response curves from the full MaxEnt model (other predictors held at their mean values), whereas blue dashed lines show response curves from univariate (single‐predictor) models. The *x*‐axis indicates predictor values, and the *y*‐axis shows the logistic output (predicted habitat suitability).

Response curves (Figure 4) indicated a complex relationship between suitability and temperature seasonality. In the univariate (single‐variable) response curve, suitability showed a sharp, pulse‐like peak in temperature seasonality (Bio4) from approximately 450 to 500 corresponding to a standard deviation of 4.5°C–5.0°C, consistent with the relatively mild seasonality of southwest China (e.g., Yunnan). However, in the marginal response curve, the suitability value did not decline markedly and remained relatively stable (0.3) once temperature seasonality (Bio4) exceeded 500 (Figure 4). This pattern supports a dual environmental response that *C. vulgare* can persist in mild, low‐seasonality areas, while also tolerating strongly seasonal, highly continental climates (cold winters and hot summers) in inland northwest China.

### 3.3 Potential Distribution of *C. vulgare* Under Current Climate Scenarios in China

**FIGURE 5:**
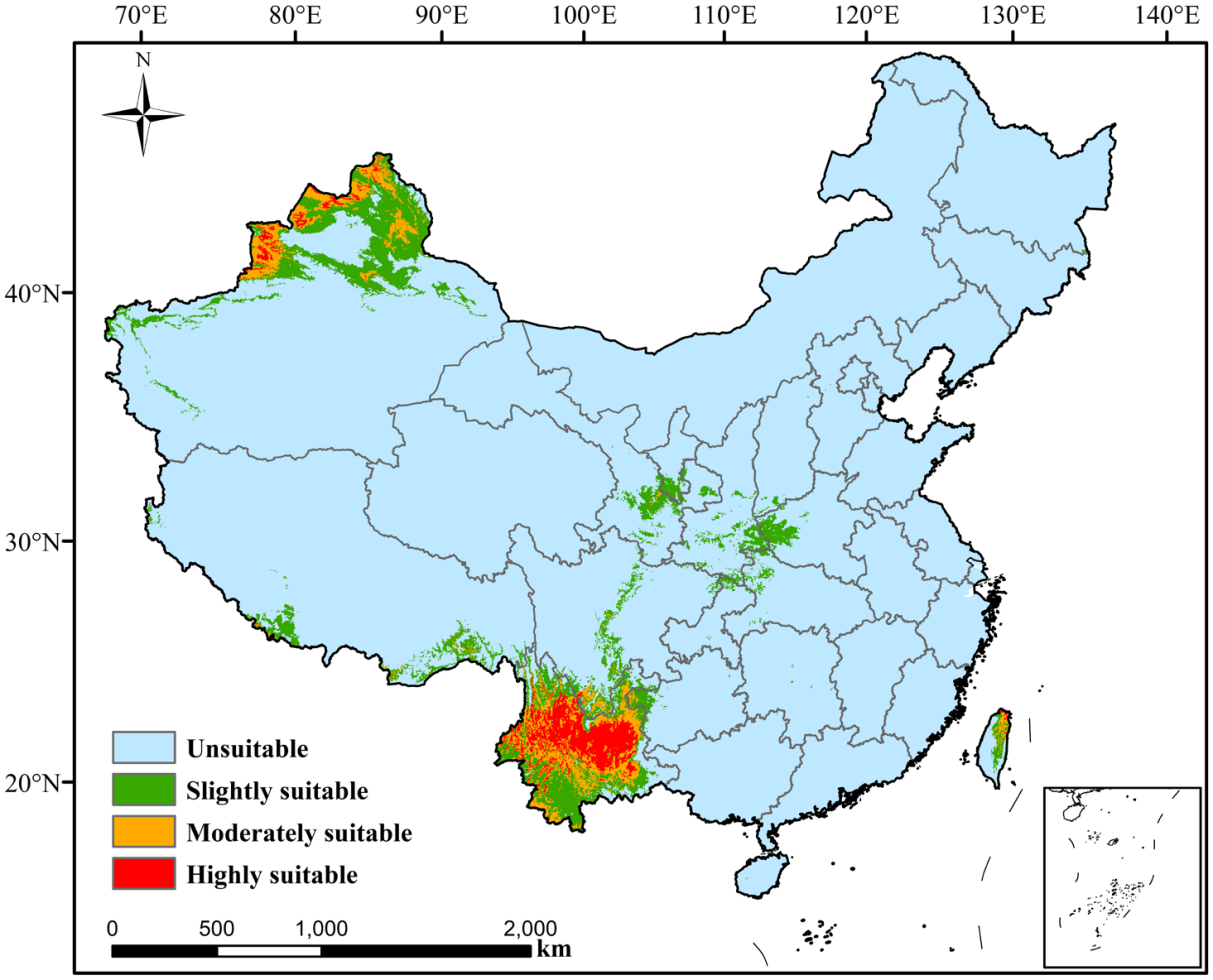
Potential distribution of *C. vulgare* in China under current climate conditions (the base map was applied from the Standard Map Service of the Ministry of Natural Resources, China, Approval Number: GS(2024)0650).

Under the current climatic conditions, the potential suitable areas of *C. vulgare* in China exhibit a multicentric pattern, with hotspots concentrated in southwest China (Yunnan and Guizhou provinces), northwest China (northern Xinjiang), and Taiwan (Figure 5). The total suitable area is estimated at 1.1491 × 10^6^ km^2^, accounting for 11.97% of China's land area. High‐suitability areas cover approximately 1.555 × 10^5^ km^2^ (1.62% of China's land area) and show a pronounced patchy, aggregated distribution, mainly in central–southern Yunnan (the core of the Yunnan–Guizhou Plateau), northern Xinjiang (specifically the Ili River Valley and the southern margin of the Junggar Basin), and the central mountain range of Taiwan.

Moderate‐suitability areas occupied 2.755 × 10^5^ km^2^ (2.87% of China's land area), largely surrounding high‐suitability patches, and was mainly distributed across eastern Yunnan–western Guizhou, southern Sichuan Province (Liangshan Prefecture), and the peripheral zones adjacent to the high‐suitability areas in northern Xinjiang. Low‐suitability areas covered 7.181 × 10^5^ km^2^ (7.48% of China's land area) and was more extensive but fragmented, occurring primarily in southern Gansu, southern Shaanxi (the Qinling Mountains), the margins of the Sichuan Basin, river valleys in southeastern Tibet, and scattered locations across other parts of central China.

### 3.4 Changes in Potential Distribution and Range of *C. vulgare* in China Under Future Climate Scenarios

**FIGURE 6:**
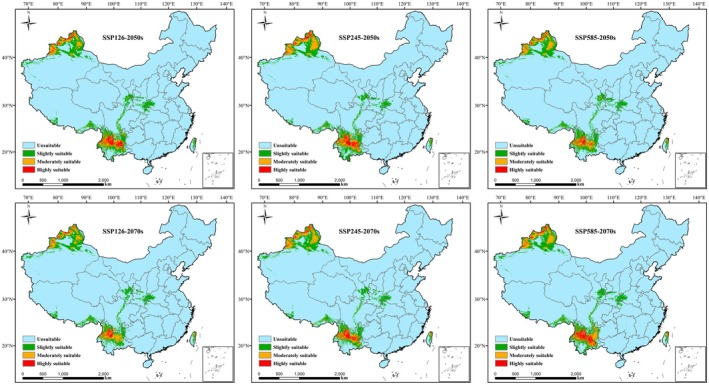
Geographic distribution of potential suitable areas for *C. vulgare* under future climate scenarios in China.

**FIGURE 7:**
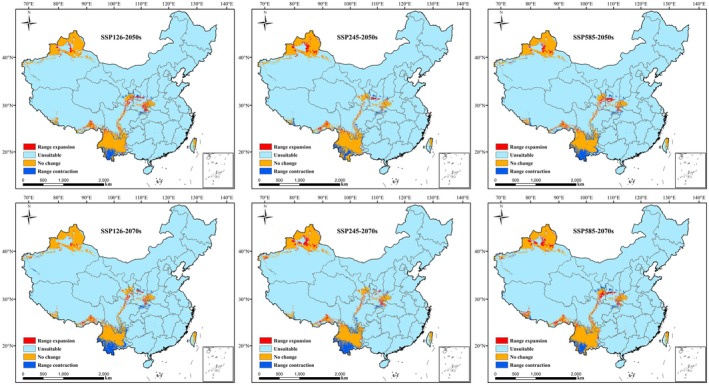
Changes in the geographic distribution of potential suitable areas for *C. vulgare* under future climate scenarios in China.

Compared to the current climate scenario, the potential suitable areas for *C. vulgare* in China under future climate scenarios presented a general pattern characterized by an overall contraction accompanied by localized fluctuations (Figures 6 and 7). Model predictions indicated that the species' range expansion rate varied between 0.81% and 1.46%, while the range loss rate varied between 1.15% and 1.79% (Table 5).

**TABLE 5:** Percentage of grid cells showing changes in habitat suitability for *C. vulgare* under future climatic conditions.

Species	Periods	SSPs	Increased	Decreased	Unchanged
*C. vulgare*	2050s	126	1.13%	1.20%	7.52%
		245	1.05%	1.15%	7.58%
		585	1.02%	1.71%	7.01%
	2070s	126	0.81%	1.79%	6.94%
		245	1.21%	1.67%	7.06%
		585	1.46%	1.21%	7.52%

Notably, with the exception of the SSP585‐2070s scenario, the rate of area suitability loss exceeded the rate of suitable area expansion in all other climate scenarios, indicating that the species faces a risk of net area suitability loss in the future (Table 5). Specifically, the contraction trend was most pronounced under the SSP126‐2070s scenario, with a net loss rate of approximately 0.98%. Conversely, under the SSP585‐2070s scenario, *C. vulgare* exhibited a unique shift from net loss to net gain, where the expansion rate (1.46%) slightly exceeded the loss rate (1.21%).

Projections for the 2050s and 2070s under SSP126, SSP245, and SSP585 (Figure 6) suggested that the spatial pattern of suitability remained broadly consistent with the current distribution. Core suitable areas persisted in the key regions of southwest China, northern Xinjiang, and Taiwan, with no evidence of large‐scale spatial relocation Specifically, **t**he spatially stable suitable areas (i.e., the “unchanged” areas derived from the binary change analysis in Table 5) consistently accounted for 6.94% to 7.58% of China's total land area across all scenarios, indicating relatively high climatic stability of the core distribution pattern. Core suitable areas persisted in the key regions of southwest China, northern Xinjiang, and Taiwan, with no evidence of large‐scale spatial relocation.

### 3.5 Centroid Migration of Potential Suitable Areas for *C. vulgare*

**FIGURE 8:**
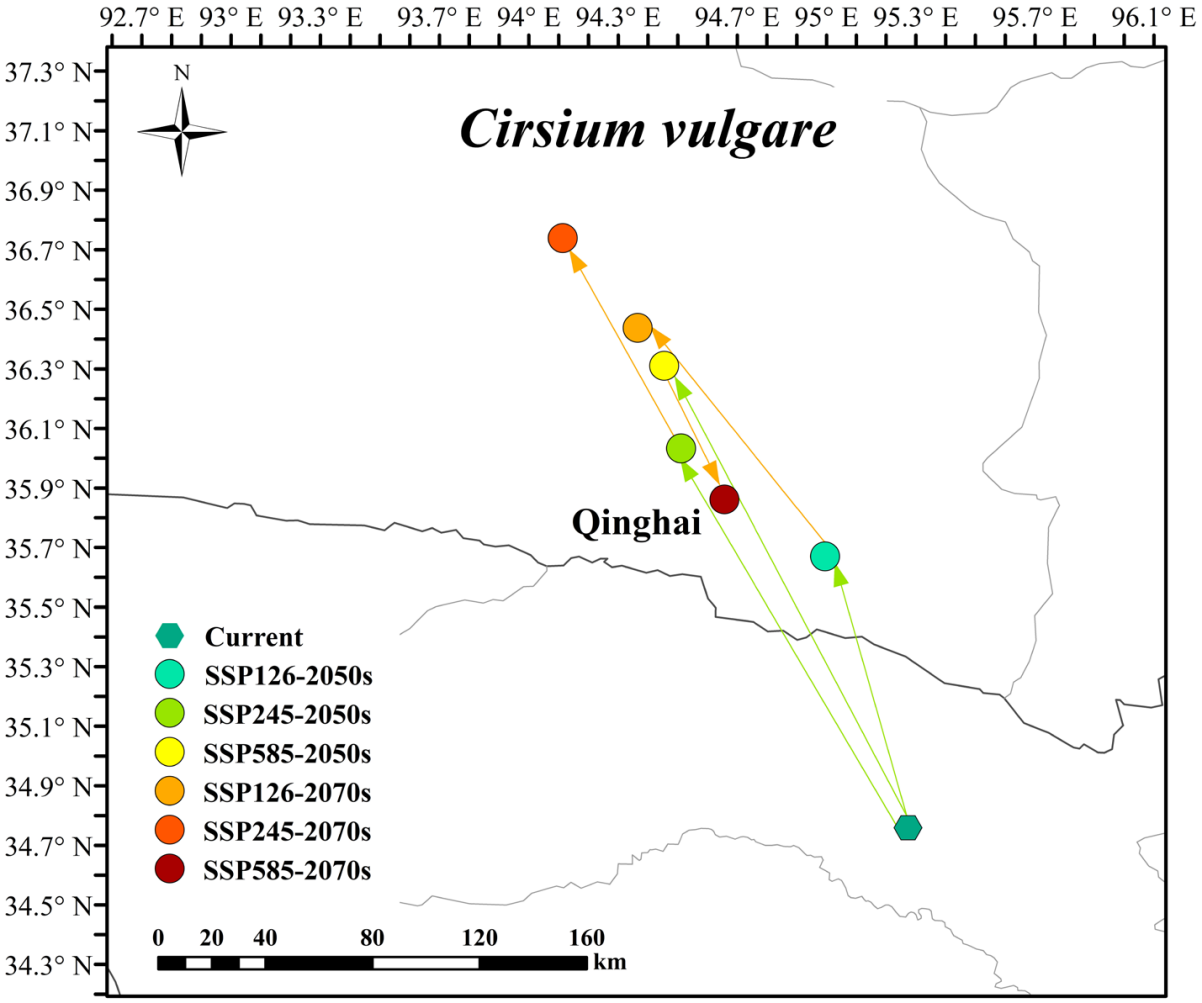
Centroid migration of *C. vulgare* under future climate scenarios in China.

Under future climate scenarios, the range for *C. vulgare* showed pronounced long‐distance, directional shifts in the centroid of suitable areas, with a non‐linear trajectory characterized by northwestward displacement followed by a late‐stage retreat (Table 6, Figure 8). Under the current climate, the centroid was located in the high‐elevation headwaters region of the Yellow River in Madoi County, Qinghai Province (95.27° E, 34.76° N). Across all scenarios in the 2050s, the centroid shifted markedly toward the northwest; the largest displacement occurs under SSP585 in the 2050s (180.2 km^2^), relocating to the southern margin of the Qaidam Basin near Golmud, Qinghai (94.45° E, 36.31° N). By the 2070s, however, the centroid under SSP585 shifted back toward its current position, with the displacement shrinking to 50.4 km^2^ (94.66° E, 35.86° N). This back‐and‐forth movement suggests that, under late‐stage extreme warming, marginal areas along the inland northwestern front may lose suitability due to excessive warming and drying, leading to a reorienting of the distribution center toward the southeast.

**TABLE 6:** Centroid coordinates and migration distances of potential suitable areas for *C. vulgare* under current and future climate scenarios.

Species	Climate scenario	Longitude (°E)	Latitude (°N)	Migration distance (km)
*C. vulgare*	Current	95.274	34.759	—
	SSP1‐2.6 (2050s)	94.994	35.671	99.253
	SSP2‐4.5 (2050s)	94.510	36.034	152.222
	SSP5‐8.5 (2050s)	94.454	36.312	180.207
	SSP1‐2.6 (2070s)	94.365	36.439	100.061
	SSP2‐4.5 (2070s)	94.113	36.740	82.298
	SSP5‐8.5 (2070s)	94.656	35.863	50.422

Centroid dynamics also indicated a pronounced downward (inverse) elevational shift. Despite the northwestward displacement, the centroid elevation of suitable areas decreased from 4922 m at present, to 3141 m near the basin margin under future scenarios. Although this predicted centroid elevation is higher than the mean elevation of occurrence records, extracted elevation data (Table 7) showed that occurrences span a wide elevational range (458–4043 m), including multiple records between 3000 and 4000 m and a maximum elevation of 4043 m in Xinjiang. This pattern supported the plausibility of high‐elevation suitability near the Qinghai–Tibet Plateau margin and suggested that the modeled high‐elevation centroid is unlikely to be a numerical artifact, but rather may reflect an unsaturated potential niche.

**TABLE 7:** Environmental characteristics and elevation of the distribution centroids of *C. vulgare* under different climate scenarios.

Species	Scenario	Mean diurnal range (Bio2) (°C)	Temperature seasonality (Bio4)	Precipitation of the driest month (Bio14) (mm)	Precipitation seasonality (Bio15) (%)	Elevation (m)
*C. vulgare*	Current	13.28	860	3	102.38	4922
	SSP126‐2050s	13.5	870	2	109.6	4533
	SSP585‐2070s	13.3	893	1	111.9	4121

### 3.6 MESS and MoD Analysis

**FIGURE 9:**
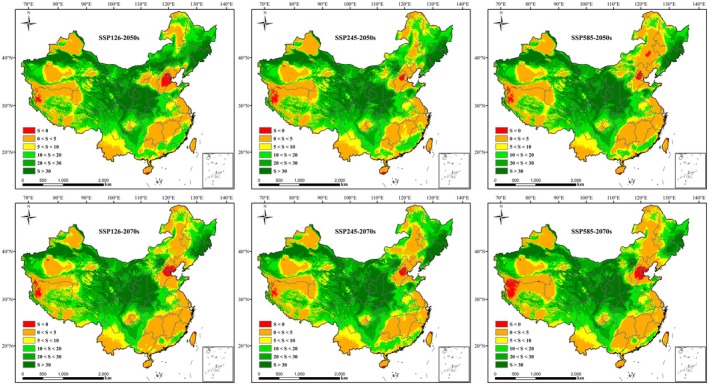
Environmental similarity and spatial distribution of novel climates under future climate scenarios.

Under all future scenarios, the climatic characteristics of the vast majority of China (> 98%) remained analogous to the calibration background (analog climates). This indicates high transferability and low extrapolation risk for future projections within the study area (Figure 9). Statistical data showed that the proportion of areas representing novel climates was extremely low, fluctuating between only 0.29% and 1.11% (Table 8), suggesting minimal risk of excessive extrapolation beyond the training data range.

**TABLE 8:** Summary of Multivariate Environmental Similarity Surface (MESS) analysis results and the Most Dissimilar (MoD) variables for *C. vulgare* under future climate scenarios.

Species	Climate scenario	Novel climates (%)	Primary limiting factor (Contribution %)	Secondary limiting factor (Contribution %)
*C. vulgare*	SSP126‐2050s	0.48	Bio15 (100%)	—
	SSP585‐2050s	0.29	Bio15 (96.8%)	Bio14 (3.2%)
	SSP126‐2070s	0.57	Bio15 (98.4%)	Bio14 (1.5%)
	SSP585‐2070s	1.11	Bio15 (99%)	Bio14 (1%)

*Note:* Bio14 = precipitation of the driest month; Bio15 = precipitation seasonality.

**FIGURE 10:**
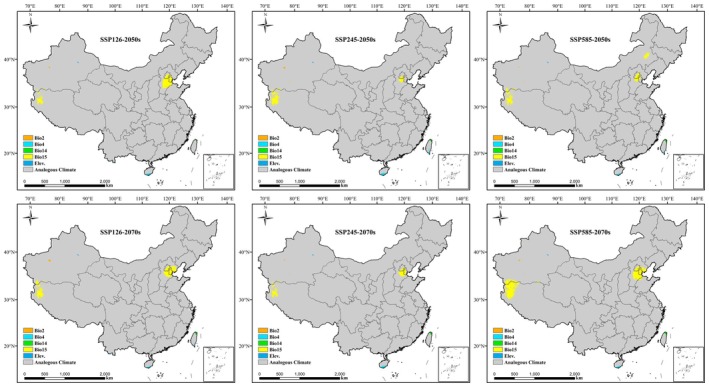
Spatial distribution patterns of the Most Dissimilar Variable (MoD) under future climate scenarios.

The MoD variable analysis further revealed the driving mechanisms causing climate anomalies in local areas. The analysis identified precipitation seasonality (Bio15) as the absolute dominant factor driving environmental novelty (Figure 10). Under the SSP126‐2050s scenario, precipitation seasonality (Bio15) accounted for 100% of the novel‐climate pixels. Even under the most drastic climate change scenario (SSP585‐2070s), precipitation seasonality (Bio15) remained the MoD variable for over 99% of the novel areas, with only a negligible fraction affected by precipitation of the driest month (Bio14).

Notably, temperature seasonality (Bio4) and Elevation were not identified as primary drivers of heterogeneity in the MoD analysis. This indicates that although future climates exhibited local fluctuations in precipitation seasonality, the future conditions of the key thermal and topographic predictors constraining the species' survival remain largely within the calibration range.

## 4 Discussion

As a newly reported invasive plant in Yunnan Province, the invasion risk and potential distribution of *C. vulgare* in China were previously poorly understood. The current study shows that *C. vulgare* has the potential to spread significantly further than its known distribution under both current and future climatic conditions. Historically considered to be primarily restricted to the temperate continental climate of Xinjiang in northwestern China, our predictive models, corroborated by recent field discoveries in the subtropical highlands, significantly update this historical baseline. The strong spatial concordance between our newly documented field occurrences in Yunnan and the model's predicted highly suitable hotspots confirms that the model effectively captures the current distribution pattern of the species. This demonstrates an expansive ecological tolerance, indicating it is not strictly confined to northwestern arid regions but poses a widespread, multicentric invasion threat across highly heterogeneous ecoregions (Davidson et al. 2011).

Our modeling demonstrated that temperature seasonality (Bio4) was identified as the dominant environmental factor shaping the potential distribution of *C. vulgare* in China, followed by precipitation seasonality (Bio15) and elevation. This result differs somewhat from broader‐scale assessments. For example, Román et al. (2021) identified mean annual temperature and precipitation during the wettest quarter as important drivers of the species' global invasion strategy. The difference between global and regional models suggests that, in China's highly heterogeneous topography, seasonal thermal variation may impose a stronger constraint on *C. vulgare* than annual mean climatic conditions.

The response curves further indicate a dual environmental response pattern. We explicitly define this pattern as a bimodal ecological strategy driven by profound phenotypic plasticity. In the mild, low‐seasonality thermal regimes of the southwestern highlands (Petitpierre et al. 2012), *C. vulgare* adopts an opportunistic life‐history strategy that maximizes rapid vegetative growth and optimal rosette overwintering (Klinkhamer and De Jong 1993). Conversely, it maintains high suitability in environments with extreme temperature seasonality (Bio4 > 1000), employing robust physiological tolerance mechanisms to endure the severe thermal fluctuations and freezing stress typical of highly continental climates in the northwest. From the perspective of Grime's CSR (Competitor, Stress‐tolerator, Ruderal) plant strategy theory (Grime 1977), this dynamic shift between Competitive‐Ruderal traits in the southwest and Stress‐tolerator characteristics in the northwest effectively broadens its realized niche. This dual pattern suggests that a single‐species distribution model, while powerful, might be capturing a fundamental niche that encompasses multiple distinct realized niches in different parts of its invaded range. While our correlative models strongly infer this macroecological pattern, future common‐garden experiments are needed to explicitly disentangle whether this is purely phenotypic plasticity or involves the development of distinct local ecotypes (Van Leeuwen 1981; Richards et al. 2006).

Geographically, this broad climatic adaptability translates to a distinct multicentric, disjunct potential distribution (Varaldo et al. 2023). The core suitable areas are highly concentrated in the southwest (Yunnan‐Guizhou Plateau), northwest (Ili Valley and the southern edge of the Junggar Basin), and Taiwan. Although geographically distant, these regions form functionally homogenous climatic areas that satisfy the species' specific thermal requirements. Crucially, our models reveal extensive potential suitable areas in Liangshan Prefecture (Sichuan), western Guizhou, and the northern foothills of the Qinling Mountains (Shaanxi). Similar to other widespread invasive species, the invasion and spread of *C. vulgare* are likely exacerbated by anthropogenic disturbances. As key ecological corridors connecting the southwest distribution center with the Central Plains hinterland (Jiang et al. 2025), these regions face a severe risk of natural and human‐mediated dispersal (Drees and Shea 2024).

Under future climate change scenarios, particularly the high‐emission SSP585‐2070s scenario, the species exhibits a substantial northwestward displacement of its habitat centroid. Unlike the commonly projected need for species to track cooler temperatures upslope, our projections for *C. vulgare* 's niche centroid indicate a rare inverse (downslope) elevational shift, dropping significantly from current high‐alpine zones (4922 m) to basin edges (3141 m)—a pattern that mirrors some observed downslope shifts in the field (Lenoir et al. 2010). However, our field surveys recorded a wide elevational span of occurrences ranging up to 4043 m, which supports the plausibility of high‐elevation suitability. This pronounced vertical reconfiguration appears to be directly linked to climatic trade‐offs. As global warming weakens seasonality in the Qinghai–Tibet Plateau hinterland (Duan et al. 2017), *C. vulgare* undergoes spatial displacement to compensate for changes in seasonal regimes. It appears to track stronger continental climates, migrating toward the edges of the Qaidam Basin where Temperature Seasonality is more intense, despite increased aridity. This indicates that maintaining specific seasonal thermal regimes likely outweighs absolute moisture requirements, demonstrating the species' strong capacity for apparent niche shifts under extreme environmental gradients (Santamarina et al. 2023).

The reliability of these ecological and management projections is underpinned by rigorous model optimization. Although the optimized MaxEnt model achieved high discriminatory performance and the MESS/MoD analyzes indicate low extrapolation risk across most of the study area (> 98%) (Cobos et al. 2019), several limitations should be acknowledged. First, the model considered only abiotic predictors, including climate and elevation, while other factors such as land use, soil type, biotic interactions, and competition with native species were not included. These factors may further influence the realized distribution of *C. vulgare* at regional or local scales (Araújo and Guisan 2006). Second, dispersal limitation was not explicitly incorporated into the model. The projections therefore indicate climatically suitable areas rather than areas that the species will necessarily colonize. The potential northwestward corridor toward the Qaidam Basin and Xinjiang should be interpreted as a possible dispersal pathway rather than a confirmed migration route. Although *C. vulgare* produces wind‐dispersed seeds, actual colonization will still depend on dispersal distance, landscape connectivity, and human‐mediated transport (Sinclair et al. 2010; Zheng, Zhang, et al. 2026). Third, future projections were based on a single GCM, BCC‐CSM2‐MR. Although this model is suitable for the objectives of this study, differences among GCMs may affect future distribution predictions, and future studies should consider multi‐GCM ensemble approaches to better quantify this uncertainty (Buisson et al. 2010).

The timely and accurate assessment of suitable habitats allows jurisdictions to plan early warning and preventative measures. Based on these invasion dynamics, a highly specific, integrated management strategy is recommended. First, the directional shift of *C. vulgare* suggests that major ecological and transportation corridors—such as those connecting southern Qinghai toward the Qaidam Basin, and the linking routes in Liangshan and the Qinling Mountains—may serve as primary pathways for human‐mediated dispersal. Enhanced quarantine, roadside monitoring, and early detection efforts are therefore highly recommended along these key corridors.

Second, because the Yunnan‐Guizhou Plateau and the Ili River Valley are projected to remain consistently suitable, representing approximately 7% of China's total land area and acting as stable climate refugia, broad‐scale eradication there may be unfeasible. Management in these strongholds should instead prioritize targeted physical and chemical control at agricultural interfaces strictly prior to the flowering stage to deplete local seed banks. Finally, rigorous agricultural sanitation protocols (e.g., screening forage seeds and cleaning machinery) must be enforced to mitigate long‐distance dispersal across these disjunct centers.

## 5 Conclusions

To the best of our knowledge, this study provides the first assessment of the potential distribution of *C. vulgare* in China using a parameter‐optimized MaxEnt model and multi‐source occurrence data. The model achieved a high mean AUC of 0.971, and MESS analysis indicated extremely low extrapolation risk (< 1.11%), demonstrating excellent discriminatory performance in the predictions. Temperature Seasonality was identified as the dominant environmental factor. Facilitated by a dual environmental response pattern that adapts to both the mild climate of the southwest and the extreme climate of the northwest, *C. vulgare* has formed a multicentric distribution pattern. Under climate change scenarios, the species exhibits a rare downslope elevational shift that reveals a unique mechanism of spatial displacement to compensate for changes in seasonal regimes, characterized by a tendency to track the edges of the Qaidam Basin where seasonal fluctuations are higher. Although the suitable area shows an overall contraction due to climate change, the Yunnan‐Guizhou Plateau and the Ili River Valley, serving as stable refugia, should be designated as primary control zones. This study elucidates the invasion pattern of *C. vulgare* and provides a scientific basis for understanding the mechanisms of niche shifts in high‐altitude invasive plants in response to climate change.

## Author Contributions

**Rongtao Hu:** data curation (equal), formal analysis (equal), methodology (equal), visualization (equal), writing – original draft (equal). **Wei Zhang:** data curation (equal), formal analysis (equal), methodology (equal), writing – original draft (equal). **Zhijie Wang:** data curation (equal), investigation (equal), writing – original draft (equal). **Qiurui Li:** investigation (equal). **David Roy Clements:** conceptualization (equal), writing – review and editing (equal). **Michael Denny Day:** writing – review and editing (equal). **Shicai Shen:** conceptualization (equal), data curation (equal), funding acquisition (equal), investigation (equal), project administration (equal), supervision (equal), writing – original draft (equal).

## Funding

This research was supported by the National Key Research and Development Program of China (2024YFC2607600), Yunnan Fundamental Research Project (202501AS070027), Yunnan Provincial Agricultural Basic Research Joint Special Project (202401BD070001‐019), National Natural Science Foundation of China (31960569), Key Research and Development Program of Yunnan Province (202103AF140007, 202203AE140008), and Ten Thousand Talent Program (Young Top‐Notch Talent) of Yunnan Province (YNWR‐QNBJ‐2018‐201).

## Conflicts of Interest

The authors declare no conflicts of interest.

## Data Availability

All data generated or analyzed during this study are included in this published article.
